# Temporal Features-Fused Vision Retentive Network for Echocardiography Image Segmentation

**DOI:** 10.3390/s25061909

**Published:** 2025-03-19

**Authors:** Zhicheng Lin, Rongpu Cui, Limiao Ning, Jian Peng

**Affiliations:** College of Computer Science, Sichuan University, Chengdu 610065, China; formlesswater@126.com (Z.L.); cuirongpu@126.com (R.C.); nlm@scu.edu.cn (L.N.)

**Keywords:** temporal feature, vision retentive network, Manhattan self-attention, echocardiography, image segmentation, deep learning

## Abstract

Echocardiography is a widely used cardiac imaging modality in clinical practice. Physicians utilize echocardiography images to measure left ventricular volumes at end-diastole (ED) and end-systole (ES) frames, which are pivotal for calculating the ejection fraction and thus quantitatively assessing cardiac function. However, most existing approaches focus on features from ES frames and ED frames, neglecting the inter-frame correlations in unlabeled frames. Our model is based on an encoder–decoder architecture and consists of two modules: the Temporal Feature Fusion Module (TFFA) and the Vision Retentive Network (Vision RetNet) encoder. The TFFA leverages self-attention to learn inter-frame correlations across multiple consecutive frames and aggregates the features of the temporal–channel dimension through channel aggregation to highlight ambiguity regions. The Vision RetNet encoder introduces explicit spatial priors by constructing a spatial decay matrix using the Manhattan distance. We conducted experiments on the EchoNet-Dynamic dataset and the CAMUS dataset, where our proposed model demonstrates competitive performance. The experimental results indicate that spatial prior information and inter-frame correlations in echocardiography images can enhance the accuracy of semantic segmentation, and inter-frame correlations become even more effective when spatial priors are provided.

## 1. Introduction

Cardiovascular disease (CVD) is one of the leading causes of mortality worldwide, with consistently high incidence and death rates. In 2021, cardiovascular disease caused 20.5 million deaths, accounting for almost one-third of all deaths [1]. The left ventricle (LV) function is closely related to overall cardiac function. Therefore, an accurate assessment of left ventricular function is critical for the diagnosis, treatment, and prognosis of cardiovascular diseases [2,3,4].

Echocardiography, a non-invasive and cost-effective cardiac imaging modality, is widely used for left ventricular function assessments. It visually reflects the anatomical structure of the left ventricle and enables both qualitative analysis and quantitative assessment of its function through the ejection fraction [5]. However, accurately segmenting the left ventricle in the end-diastolic (ED) and end-systolic (ES) frames (key frames) is a prerequisite for calculating the ejection fraction [6]. This segmentation process is labor-intensive and susceptible to subjective interpretation by clinicians. Therefore, there is a growing demand for automated segmentation of the left ventricle to enhance efficiency and reduce bias in the assessment of cardiac function.

**Figure 1:**
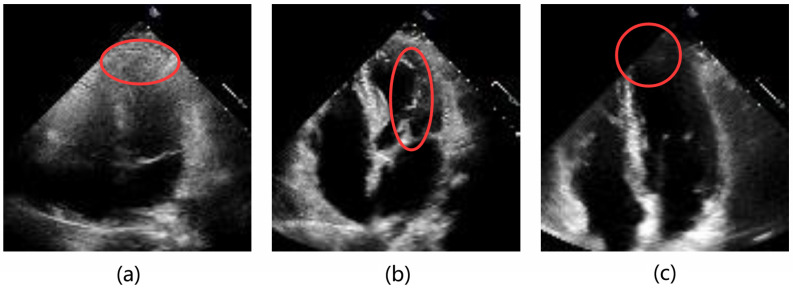
Several challenges in echocardiography image segmentation: (**a**) Blurred contours due to speckle noise. (**b**) Ambiguous contours caused by papillary muscle. (**c**) Contours out of the field of view.

In recent years, with the advancement of deep learning, convolutional neural networks (CNNs) have achieved remarkable success in natural image segmentation and extended their applications to medical imaging across various modalities, including CT [7], MRI [8], and ultrasound [9]. This indicates that deep learning has tremendous potential and broad applicability in computer-automated diagnostic methods. However, as shown in Figure 1, there are still several challenges in automated echocardiography image segmentation (see Figure 1): (1) There is a significant amount of noise and artifacts in echocardiography images, which cause the boundaries of the left ventricle (LV) to be blurred and hinder accurate segmentation. (2) Echocardiography images have low resolution and contrast, making it difficult to distinguish the left ventricle from other intracardiac tissues, such as papillary muscles, thrombi, and tumors, and interfere with the segmentation of the left ventricle. (3) The movement of the heart and the ultrasound probe can cause parts of the left ventricle to move out of the field of view, resulting in incomplete left ventricular shapes in the images. In summary, these factors render the left ventricle’s contours ambiguous and objectively limit the segmentation performance of existing methods.

Since the inception of U-Net [10] and its remarkable success in medical image segmentation, encoder–decoder architectures powered by CNNs have become the norm for semantic segmentation tasks. However, due to the limitations of receptive fields, CNNs are unable to effectively capture long-range dependencies within images. The Transformer [11], originally applied to natural language processing tasks, is a sequence prediction model. The core of the Transformer lies in its multi-head attention mechanism, which possesses a global receptive field, enabling it to capture long-range dependencies. Following the introduction of the Vision Transformer [12], many studies [13,14,15,16,17] began to explore hybrid architectures that combine CNNs and Transformers. These architectures leverage the Transformer to extract global correlations, complementing the local receptive fields of CNNs. In the task of echocardiography image segmentation, CNNs, with their local receptive fields, may struggle to provide accurate predictions when confronted with ambiguous contour features. The integration of the Transformer into CNN models introduces global correlations, allowing the model to more accurately perceive the contours of the left ventricle while maintaining consistency in the shape of the segmented region. This characteristic significantly enhances the model’s robustness against the ambiguous contours caused by speckle noise and the movement of the heart or the ultrasound probe.

**Figure 2:**
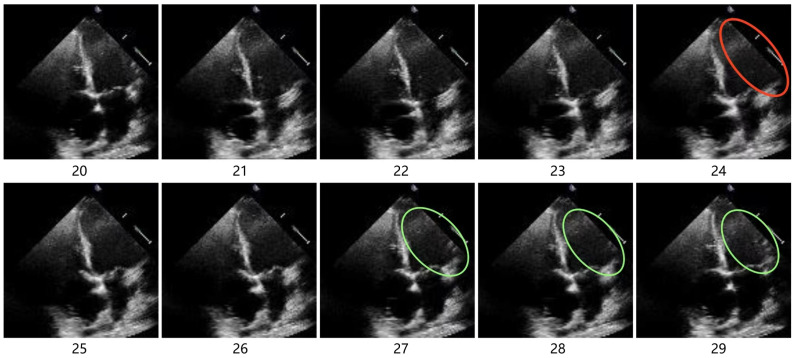
A clip that contains an end-diastolic frame (frame 24).

In addition to the global correlations within a single echocardiography image, there also exist inter-frame correlations between the frames of the echocardiography images. Taking the video frames illustrated in Figure 2 as an example, frame 24 is the ED frame. In the red-circled area, the contour of the left ventricle extends beyond the field of view and cannot be determined. In frames 27 to 29, as the heart moves, the previously indeterminate boundary (green-circled area) reappears in the field of view, allowing for a rough estimate of the ambiguous portions of the contour in frame 24. Thus, there is evidence suggesting that inter-frame correlation also plays a crucial role in helping the model overcome the challenge of the ambiguous contours. However, the existing hybrid CNN–Transformer methods are primarily designed for the segmentation of single-frame images without considering the inter-frame correlations inherent in echocardiography videos.

On the other hand, the self-attention mechanism in the Transformer [11], while effective, has quadratic computational complexity, leading to high computational costs. To mitigate this, the Retentive Network (RetNet) [18] features a novel multi-scale retention mechanism, which replaces the multi-head self-attention in Transformers, and introduces an explicit decay of information, featuring linear computational complexity. Based on this, Fan et al. [19] successfully extended the time decay mechanism of RetNet to two-dimensional space by representing explicit spatial prior information through the Manhattan distance, thereby making RetNet applicable to image data and achieving superior performance to Transformers with reduced computational complexity.

In summary, to overcome the contour ambiguity issues caused by noise, cardiac tissue, and motion factors in echocardiography images, existing methods incorporate global correlations through the self-attention mechanism of Transformers to maintain the shape consistency of segmentation results. We believe that inter-frame correlations in echocardiograms can also help mitigate boundary blurring, and the emergence of RetNet offers a lower computational cost and more effective alternative to Transformers. Thus, we propose an automatic segmentation model. This model effectively utilizes the inherent inter-frame correlations, fusing temporal features to enhance the segmentation of ambiguous regions. The Vision Retentive Network, serving as the encoder, offers superior feature extraction capabilities and lower computational complexity compared to Transformers.

Our contributions can be summarized as follows:

(1) We propose the Temporal Feature Fusion Module (TFFM) based on temporal–channel self-attention. It fuses the temporal and channel dimensions and applies self-attention to the fused dimension to capture the inter-frame correlations in echocardiography images. Additionally, we introduce a channel aggregation module that extracts complementary interaction information between channels to reallocate features of the fused channels. This module summarizes the features of multiple frames and enhances the features of ambiguous regions in key frames caused by noise and artifacts.
(2) We introduce the Vision RetNet as the encoder in echocardiography segmentation. Vision RetNet constructs a spatial decay matrix by introducing explicit spatial prior information through the Manhattan distance, extending the retention mechanism used for one-dimensional sequences to two-dimensional images. Therefore, Vision RetNet can learn spatial correlations in the image effectively.
(3) We propose an end-to-end video sequence segmentation model that combines the advantages of the two modules mentioned above, achieving competitive performance compared to existing models on the EchoNet-Dynamic dataset and the CAMUS dataset.

## 2. Related Work

### 2.1. Semantic Segmentation in Cardiac Images

With the extensive application of deep learning across multiple modalities of medical images (such as CT, MRI), some studies began to explore the use of deep learning methods for semantic segmentation in cardiac images. Leclerc et al. released the CAMUS (Cardiac Acquisitions for Multi-Structure Ultrasound Segmentation) dataset [20], which includes images in both apical 4 chamber (A4C) and apical 2 chamber (A2C) views from 500 patients. For each patient in the training dataset, images and expert annotations of the left ventricle, myocardium, and left atrium are provided for the ED and ES frames of the cardiac cycle. Leclerc et al. compared the performance of several medical image semantic segmentation models, including UNet [10] and UNet++ [21], on the CAMUS dataset. Furthermore, they introduced LU-Net [22], a two-stage segmentation network wherein the first stage is a Region Proposal Network for predicting bounding boxes of regions of interest, and the second stage is a pure U-Net for semantic segmentation. Moradi et al. proposed MFP-Unet [23], which incorporates dilated convolutions into the Unet architecture to increase the receptive field of the CNN. Additionally, it applies Feature Pyramid Networks (FPNs) [24] in the decoder part, extracting feature maps from each decoder layer for the final pixel-wise classification. Ali et al. proposed the Res-U network [25], utilizing ResNet-50 as the encoder. They modified the ResNet architecture specifically for echocardiography images by retaining the input data from the initial layer and propagating it to each block in every layer, with the convolutional results being accumulated with the input of each block. Inspired by ConvNext, Zhang et al. [26] incorporated an upsampling module into the ConvNext architecture and combined it with the encoder–decoder structure of UNet, proposing a segmentation network with strong generalization capabilities for heart MRI image segmentation. The model introduces an attention mechanism in the bridging path, innovatively generating probability distributions as attention weights from both the encoder and decoder sides through linear and nonlinear transformations, thereby emphasizing features in the regions of interest.

Ouyang et al. introduced a large-scale echocardiogram video dataset called EchoNet-Dynamic [27], which contains 10,030 video sequences. This dataset includes left ventricle annotations provided by experts in the form of coordinate points. They utilized DeeplabV3 [28] to perform left ventricle semantic segmentation and ejection fraction prediction on the EchoNet-Dynamic dataset. Several studies were conducted on the EchoNet-Dynamic dataset. PLANet [29] introduces a pyramid local attention module that captures supportive information from compact and sparse contexts to enhance features. It guides the learning with explicit supervision signals to promote prediction consistency of adjacent pixels. Transbridge [30] is a lightweight architecture that bridges the encoder and decoder using a Transformer. It reduces the number of parameters in the embedding layer and the size of the token sequence by employing dense patch division and shuffled group convolution. EchoGraphs [31] utilizes a graph convolutional network to learn the representation of the cardiac shape based on local appearance of each keypoint and global spatial and temporal structures of all keypoints combined for the regression prediction of keypoint coordinates. MAEF-Net [32] incorporates a dual-attention layer between every layer of the encoder and decoder. This dual-attention layer consists of two branches, spatial attention and channel attention, which guide the network to capture cardiac features and suppress noise. The decoder part introduces a deep supervision mechanism, and the final prediction is fused from the outputs of each layer. Chen et al. [33] proposed a weakly supervised encoder–decoder model, UDeep, where the encoder utilizes Separated Xception as the backbone, combined with the Atrous Spatial Pyramid Pooling module to extract multi-scale semantic features. In the decoder section, multiple upsampling fusion modules are employed to integrate features from different levels. Notably, UDeep introduces an Euclidean distance-based pseudo-segmentation penalty loss function, which imposes varying degrees of penalty for pseudo-segmentation.

### 2.2. Medical Image Segmentation with Inter-Frame Correlations

In recent years, some studies focused on leveraging multi-frame or inter-frame information from medical image sequences to enhance the correlated features. Ahn et al. [34] proposed a multi-frame attention network, where multi-frame attention mechanisms can learn correlations between the key frames and subsequent frames, respectively, leveraging the highly correlated spatio-temporal features for augmenting the segmentation performance. MedSeq [35] follows the “localization-then-refinement” paradigm, designing a cross-frame attention module to learn correlations among frames for localizing target regions. For refinement, MedSeq utilizes a boundary-aware Transformer to improve the segmentation of boundary patches. Video-SwinUNet [36] introduced a novel temporal context module to blend features from neighboring frames to enhance temporal consistency and combines it with a Swin Transformer in the encoder to enhance global context understanding. For echocardiography images, Li et al. [37] proposed a myocardial segmentation model for quantitative myocardial contrast echocardiography. This model uses a U-Net-based architecture and incorporates convolutional long short-term memory (LSTM) in the encoder to capture inter-frame correlations in video sequences. Inspired by recurrent neural networks, the model adopts a bidirectional training strategy, which enhances temporal coherence by leveraging temporal information from both forward and backward directions. CLA-U-Net [38] employs a similar architecture on the EchoNet-Dynamic, except that it replaces the skip connections in U-Net with a channel attention mechanism to enhance the desired feature signals and suppress noise.

## 3. Methods

**Figure 3:**
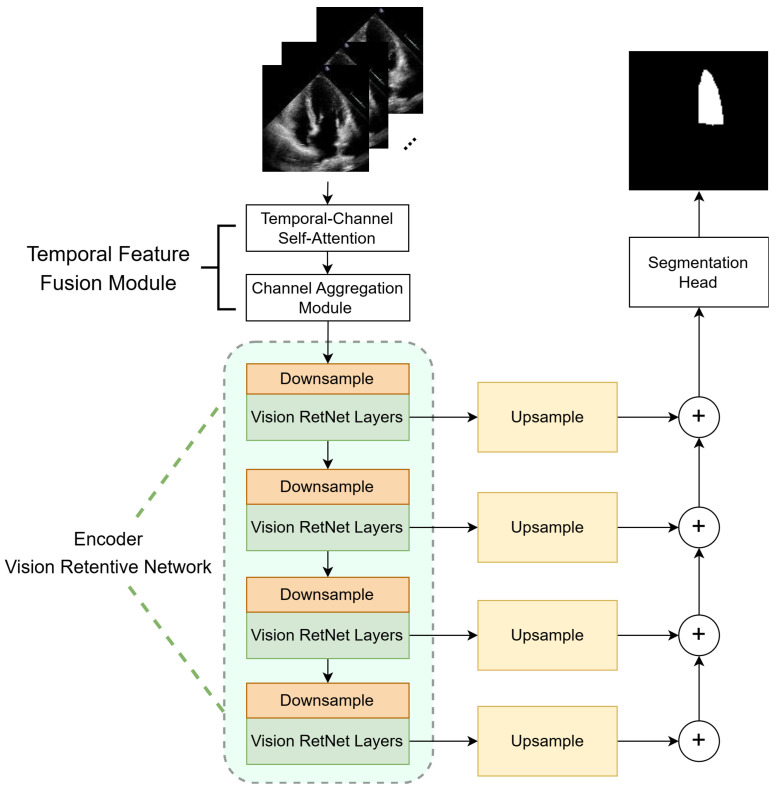
The overview of the model proposed in this paper.

The model proposed in this paper is based on the classic encoder–decoder architecture, which consists of three main components (see Figure 3): the Temporal Feature Fusion Module (TFFM), the Vision Retentive Network encoder, and the Feature Pyramid Networks decoder. The Temporal Feature Fusion Module employs self-attention to learn inter-frame correlations in the sequence. The Vision RetNet encoder leverages the Manhattan distance to introduce explicit spatial prior information for feature extraction. The Feature Pyramid Networks decoder upsamples the multi-scale features generated by the Vision RetNet to the same resolution, then employs element-wise addition on them, thereby integrating semantic information from deeper networks with detailed information from shallower networks. The final segmentation result is obtained through a segmentation head.

### 3.1. Temporal Feature Fusion Module

#### 3.1.1. Temporal–Channel Self-Attention (TCSA)

**Figure 4:**
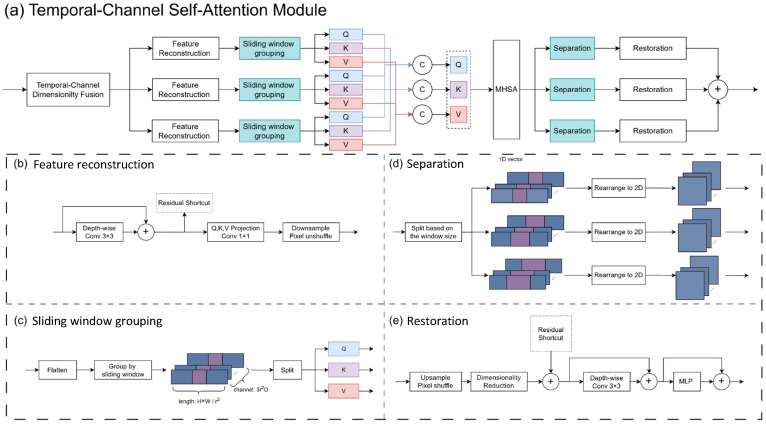
The overall architecture of the Temporal Feature Fusion Module and its details.

The self-attention mechanism assists the model in capturing inter-frame correlations from temporal features, which in turn enhances its comprehension of the left ventricular morphology. Consider the input sequence as $X\in R^{T\times C\times H\times W}$, where *T* is the number of frames in the sequence, *C* is the number of channels, and H×W is the spatial resolution of each frame. We concatenate each frame in the sequence along the channel dimension to fuse the channel and temporal dimensions and obtain $X\in R^{TC\times H\times W}$. To obtain varying temporal–channel receptive fields, we set three windows of different sizes, reconstructing and grouping the features, respectively. Specifically, as illustrated in Figure 4b, *X* is embedded in a high-dimensional space and the spatial dimension information is compressed into the channel dimension. After that, we use a linear projection layer to obtain query (*Q*), key (*K*), and value (*V*) vectors, respectively. This process not only enhanced the feature representation capacity but also reduced computational complexity, ultimately producing $X\in R^{3r^{2}D\times \frac{H}{r}\times \frac{W}{r}}$. Here, *r* is the spatial compression ratio and *D* denotes the dimensionality of the embedded space. The *Q*, *K*, and *V* vectors are sequentially arranged along the temporal–channel dimension of *X*.

After feature reconstruction, the spatial dimensions of *X* are flattened into one dimension, and features are grouped along the temporal–channel dimensions using a sliding window according to the given window size $w_{i}$ (see Figure 4c). The output of the grouping is $X\in R^{3r^{2}D\times w_{i}\times \left(\frac{HW}{r^{2}}\right)}$. By concatenating the results of the three groupings, we obtain $X\in R^{3r^{2}D\times \sum_{i=1}^{3}w_{i}\times \left(\frac{HW}{r^{2}}\right)}$. Then, according to the self-attention formula in Equation (1), the vectors *Q*, *K*, and *V* are extracted from *X* to calculate the temporal–channel attention matrix. This process captures the correlation in the temporal–channel dimension of the input features *X*, enabling the model to perceive the connections between multiple frame features, thereby capturing inter-frame correlations.

(1) $$\mathrm{SA}\left(X\right)=\mathrm{softmax}\left(QK^{T}\right)V$$

After computing the correlation, the grouped features are restored to their original size in two steps (see Figure 4d,e). In the first step, the features are separated according to the window size used for grouping, transforming the flattened 1D sequence back into a 2D image. In the second step, the features are reduced from high-dimensional space to the original T×C dimensions and restored to the original resolution of the image. The restoration results from different window lengths are summed to obtain the final output $X_{TCSA}\in R^{T\times C\times H\times W}$ of this module.

#### 3.1.2. Channel Aggregation Module (CA)

**Figure 5:**
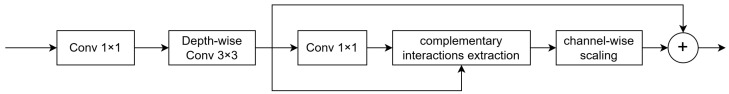
The structure of the channel aggregation module.

To bolster the model’s robustness against ambiguity in echocardiography images, we introduce a channel aggregation module [39] (see Figure 5). This module extracts complementary interaction information along the time–channel dimensions to aggregate features, thereby enhancing the segmentation of ambiguous regions. This module takes the output of the temporal–channel self-attention $X_{TCSA}\in R^{T\times C\times H\times W}$ as its input. It then aggregates the input features along the temporal–channel dimensions. The process begins with a 1 × 1 convolution to expand feature representation, where *r* is the rate of expansion. Subsequently, a 3 × 3 depth-wise convolution is applied to capture distinct feature expressions within each channel and obtain $Y\in R^{rTC\times H\times W}$, as illustrated in Equation (2). Following this, the feature aggregation is conducted: a 1 × 1 convolutional kernel extracts universal features across the temporal–channel dimensions $X_{u}\in R^{1\times H\times W}$, subtracting these from the temporal–channel features to derive complementary features. These complementary features encompass characteristics of regions that are ambiguous in key frames but relatively distinct in frames close to the key frames. By introducing a learnable parameter *s*, the module adaptively integrates these complementary features to refine the feature representation along the temporal–channel dimensions, thereby augmenting the model’s capabilities, as depicted in Equation (3). Finally, the enhanced features are merged with the original features through a residual connection, as shown in Equation (4).

(2) $$Y=\mathrm{GELU}({\mathrm{DWConv}}_{3\times 3}\left({\mathrm{Conv}}_{1\times 1}\left(\mathrm{Norm}\left(X_{TCSA}\right)\right)\right))$$

(3) $$\mathrm{CA}\left(Y\right)=Y+s⊙(Y-\mathrm{GELU}\left({\mathrm{Conv}}_{1\times 1}\left(Y\right)\right))$$

(4) $$X_{out}={\mathrm{Conv}}_{1\times 1}\left(\mathrm{CA}\left(Y\right)\right)+X_{TCSA}$$

### 3.2. Vision Retentive Network Encoder

The model we propose resembles a hybrid Transformer–CNN architecture; however, the Transformer is replaced by Vision RetNet. Vision RetNet is an extension of RetNet, introducing explicit spatial decay through the Manhattan distance to make RetNet available on two-dimensional (2D) image data.

#### 3.2.1. Manhattan Self-Attention

RetNet [18] proposes a retention mechanism that leverages temporal decay to represent relative distances in one-dimensional (1D) sequences, thereby introducing explicit temporal prior information into sequence modeling. Retention mechanism can be formulated as Equation (5), where *D* contains both causal masking and 1D exponential decay and ⊙ denotes the Hadamard product.

(5) $$\mathrm{Retention}\left(X\right)=\left(QK^{T}⊙D^{1d}\right)V$$

(6) $$D_{nm}^{1d}=\left\{\begin{array}{cc} \gamma^{n-m},\; & n\geq m\\ 0,\; & n<m \end{array}\right.$$

To extend the retention mechanism to 2D image data, a metric capable of representing relative spatial distances is required to construct spatial decay. Thus, the Manhattan distance is considered to represent relative spatial distances and introduce explicit spatial prior information. By considering a token as a point in 2D space, each token can be assigned a unique coordinate to represent its position in the 2D space. Suppose the positions of two tokens are $\left(x_{1},y_{1}\right)$ and $\left(x_{2},y_{2}\right)$; their spatial Manhattan distance can be expressed as $\left|x_{1}-x_{2}\right|+\left|y_{1}-y_{2}\right|$. Based on their Manhattan distance, their spatial decay can be calculated as $\gamma^{\left|x_{1}-x_{2}\right|+\left|y_{1}-y_{2}\right|}$. Generalizing to any two tokens, the spatial decay matrix is expressed as $D_{nm}^{2d}=\gamma^{\left|x_{n}-x_{m}\right|+\left|y_{n}-y_{m}\right|}$. Then, to introduce nonlinearity, a softmax function is employed, similar to self-attention. Therefore, the Manhattan self-attention can be expressed as Equation (7).

(7) $$\mathrm{MaSA}\left(X\right)=(\mathrm{Softmax}\left(QK^{T}\right)⊙D^{2d})V$$

To reduce computational complexity without compromising the spatial decay matrix based on Manhattan distance, the 2D Manhattan self-attention is decomposed into two 1D self-attentions, yielding a horizontal component $Attn_{H}$ and a vertical component $Attn_{W}$. Correspondingly, the 2D spatial decay matrix is also decomposed into two 1D spatial decay matrices, representing the horizontal and vertical distances between tokens, respectively. Thus, the decomposed Manhattan self-attention can be expressed as follows:

(8) $$Attn_{H}=\mathrm{Softmax}\left(Q_{H}{K_{H}}^{T}\right)⊙D^{H},D_{nm}^{H}=\gamma^{\left|y_{n}-y_{m}\right|}$$

(9) $$Attn_{W}=\mathrm{Softmax}\left(Q_{W}{K_{W}}^{T}\right)⊙D^{W},D_{nm}^{W}=\gamma^{\left|x_{n}-x_{m}\right|}$$

(10) $$\mathrm{MaSA}\left(X\right)=Attn_{H}{\left(Attn_{W}V\right)}^{T}$$

#### 3.2.2. Encoder Architecture

**Figure 6:**
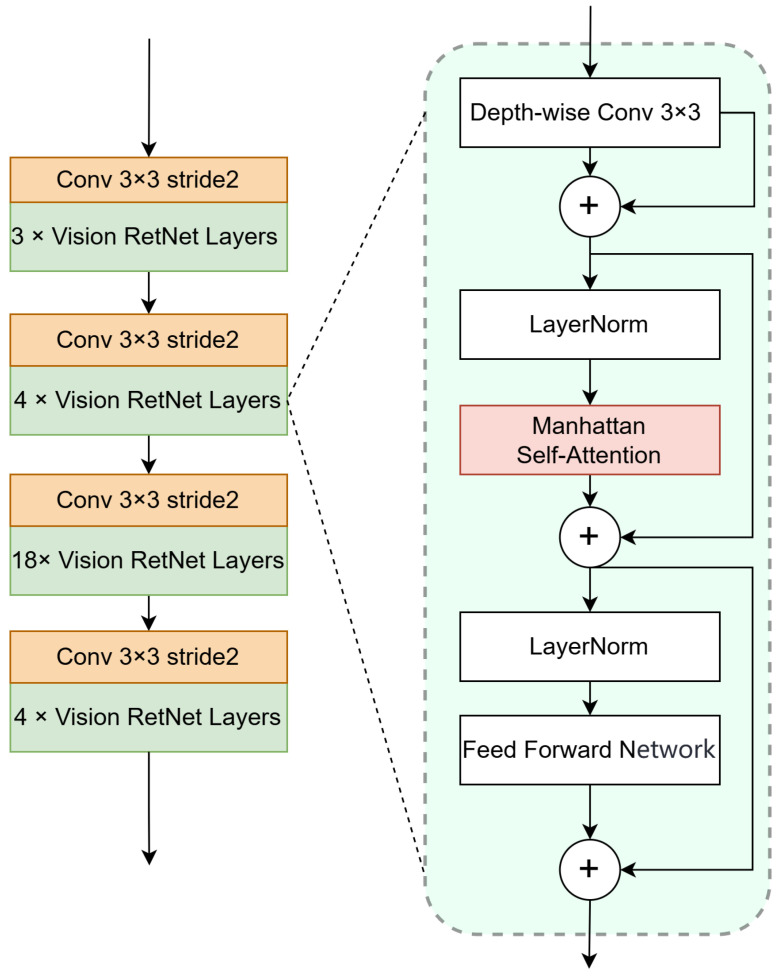
The overall architecture of the Vision Retentive Network encoder and the details of a single Vision Retentive Network layer.

Based on the Manhattan self-attention described in the previous section, a four-stage Vision RetNet is designed as the encoder for feature extraction, as shown in Figure 6. The first three stages use decomposed Manhattan self-attention, while the last stage employs the original Manhattan self-attention. Each stage consists of 3, 4, 18, and 4 layers, respectively. Each layer consists of four parts: conditional positional encoding, Manhattan self-attention, local context enhancement module, and a feed-forward network layer. Before each stage, a 3 × 3 convolution with a stride of 2 is used for downsampling.

First, conditional positional encoding is applied to the feature map. Unlike traditional positional encoding, conditional positional encoding is a translation equivariance encoding that is dynamically generated based on the neighborhood of the input token as a condition. It is produced by a 3 × 3 depth-wise separable convolution with zero-padding of 1, where the padding primarily allows boundary pixels to also perceive their absolute positions. This part can be expressed as follows:

(11) $$X=X+\mathrm{CPE}\left(X\right),\mathrm{CPE}\left(X\right)={\mathrm{DWConv}}_{3\times 3}\left(X\right)$$

After completing the positional encoding, three linear projections are applied to obtain the query, key, and value corresponding to the feature map. Manhattan self-attention is then calculated based on these three components, while the value is fed into the Local Context Enhancement module [40]. This module consists of a 5 × 5 depth-wise separable convolution with zero-padding of 2, which enhances the local expression capability. This part can be expressed as follows:

(12) X=MaSA(X)+LCE(V)

Finally, the feature map is fed into a feed-forward network to obtain the final output.

#### 3.2.3. Computational Complexity Analysis

In this section, we will provide a detailed analysis of the time complexity of the Manhattan self-attention in Vision RetNet, and compare it with the self-attention in Transformer.

Let $Q,K,V\in R^{N\times d}$, where *d* represents the embedding dimensionality and *N* denotes the number of patches. Considering only a single head, the self-attention mechanism in the Transformer can be expressed as Equation (1), and the computational complexity of the self-attention mechanism can be derived in three steps: (1) *Q* and *K* Matrix Multiplication: The calculation of the attention scores involves multiplying the *Q* matrix with the transpose of the *K* matrix, which has a time complexity of $O(N^{2}d)$. The result of the matrix multiplication will have a shape of (N,N). (2) Softmax function: The softmax function, applied to each row of the attention score matrix, has a time complexity of $O(N^{2})$. (3) Value Multiplication: The resulting attention weights, which have a shape of (N,N), are then multiplied by the matrix *V*. The time complexity of this multiplication is $O(N^{2}d)$. Thus, the overall time complexity for the single-head self-attention is $O(N^{2}d)$.

For Vision RetNet, the original Manhattan self-attention (MaSA) can be formulated as Equation (7). The key distinction from the self-attention of Transformer lies in the addition of an intermediate step between steps 2 and 3, where the output of the softmax function is element-wise multiplied with the spatial decay matrix *D* ($D\in R^{N\times N}$). The computational complexity of this step is $O(N^{2})$. Thus, the overall computational complexity is the same as the original Transformer ($O(N^{2}d)$).

However, the decomposed MaSA is different. Firstly, the input *Q*, *K*, and *V* are not tokenized. This means that $Q,K,V\in R^{H\times W\times d}$ and H×W=N. The images in both the EchoNet dataset and the CAMUS dataset satisfy H=W. Next, the attention scores in the horizontal direction are calculated according to the formula $Attn_{H}=\mathrm{Softmax}\left(Q_{H}{K_{H}}^{T}\right)⊙D^{H}$, where $Q_{H}\in R^{H\times W\times d}$ and ${K_{H}}^{T}\in R^{H\times d\times W}$. The shape of the matrix multiplication result is (H,W,W) and the computational complexity of their matrix multiplication is $O(W^{2}d)$. The complexity of the softmax function and subsequent computation with the decay matrix is related to the shape of the input data, both of which are $O(W^{2})$. So, the computational complexity of the horizontal attention scores is $O(W^{2}d)$. The process of calculating the attention scores in the vertical direction is similar, according to $Attn_{W}=\mathrm{Softmax}\left(Q_{W}{K_{W}}^{T}\right)⊙D^{H}$. The difference lies in the swap of the *H* and *W* dimensions of the input $Q_{W}$ and $K_{W}$ before the matrix multiplication. Referring to the derivation process of the complexity for horizontal attention scores, the computational complexity of the vertical attention scores is $O(H^{2}d)$.

After obtaining the attention scores in both directions, the multiplication results of the attention scores with the V matrix are computed sequentially. This process is represented by the formula $\mathrm{MaSA}\left(X\right)=Attn_{H}{\left(Attn_{W}V\right)}^{T}$, where $Attn_{W}\in R^{H\times W\times W}$, $Attn_{H}\in R^{W\times W\times H}$ and $V\in R^{H\times W\times d}$. The computational complexity of the multiplication between $Attn_{W}$ and the *V* matrix is $O(W^{2}d)$. The computational complexity of the matrix multiplication between $Attn_{W}$ and the result is $O(H^{2}d)$. As mentioned earlier, H=W and N=H×W, the overall computational complexity is O(Nd). Thus, it is evident that the computational complexity of the decomposed MaSA is linear, requiring significantly fewer computational costs compared to the quadratic complexity of standard self-attention.

## 4. Experiments

### 4.1. Dataset

The CAMUS dataset [20] consists of clinical exams from 500 patients, acquired at the University Hospital of St Etienne (Saint-Étienne Cedex 2, France). The full dataset was acquired from GE Vivid E95 ultrasound scanners (GE Vingmed Ultrasound, Horten, Norway), with a GE M5S probe (GE Healthcare, Chicago, IL, USA). A total of 400 patients were divided into a training set, with 50 patients allocated to the validation set and 50 patients to the test set. Each patient’s data consist of two image sequences, representing the left ventricle in the 2-chamber and 4-chamber views. Each image sequence captures half of the cardiac cycle, ranging from end-diastole to end-systole. Each image in the dataset is annotated and provided as a mask. Each image contains three types of annotation information: the endocardium, epicardium, and left atrium. We standardize by considering the region enclosed by the endocardium as the left ventricle, and thus only retain the annotation information for the endocardium. The size of the images is 256 × 256.

The Echonet-Dynamic dataset [27] is a large-scale publicly available echocardiogram dataset containing 10,030 apical 4-chamber echocardiogram videos. The dataset was collected from 10,030 healthy volunteers or cardiac patients, who underwent echocardiography at Stanford Health Care between 2016 and 2018. Videos were acquired by skilled sonographers using iE33 (Philips, Amsterdam, The Netherlands), Sonos (Philips, Amsterdam, The Netherlands), Acuson SC2000 (Siemens Healthineers, Malvern, PA, USA), Epiq 5G (Philips, Amsterdam, The Netherlands), or Epiq 7C (Philips, Amsterdam, The Netherlands) ultrasound machines. The collected videos were then stored in a Philips Xcelera (Philips, Amsterdam, The Netherlands) picture archiving and communication system. Only the end-diastolic and end-systolic frames in each video were annotated. After annotation, the videos were cropped and masked to remove text, identifying information and other details outside of the scanning sector. Then, the processed videos were downsampled to 112 × 112 pixels using cubic interpolation. Since the model uses video clips containing the end-diastolic and end-systolic frames as input, we performed data cleaning on the dataset, retaining only videos with at least a total of 8 frames surrounding these key frames. The cleaned dataset contains a total of 9158 videos, with 6805 videos assigned to the training set, 1187 videos to the validation set, and 1166 videos to the test set.

### 4.2. Evaluation Metrics

We use the Dice similarity coefficient (DSC) and Hausdorff Distance (HD), two commonly used metrics in image segmentation, to evaluate the proposed model. DSC is sensitive to overlap between the predicted and ground truth masks. HD is a metric that calculates the maximum distance between corresponding points on the boundary of patches belonging to the same class. Assuming the model’s predicted mask is denoted as set A and the manually annotated ground truth as set B, the DSC and HD can be formulated via Equations (13) and (14):

(13) $$\mathrm{DSC}\left(A,B\right)=\frac{2|A\cap B|}{\left|A|+|B\right|}$$

(14) $$\mathrm{HD}\left(A,B\right)=\max\left(h\left(A,B\right),h\left(B,A\right)\right),h\left(A,B\right)=\max_{a\in A}\{\min_{b\in B}||a-b||\}$$

### 4.3. Implementation Details

We implemented the proposed method with PyTorch 3.8.19, and all experiments were run on a workstation with the Intel(R) Xeon(R) CPU E5-2680 v4 (Intel, Santa Clara, CA, USA) @2.40 GHz, NVIDIA RTX 3090 GPU (NVIDIA, Santa Clara, CA, USA) and 64 G of RAM. For detailed information regarding the Python package version, please refer to Appendix A. The model employs Mean Absolute Error (MAE) as the loss function, utilizes AdamW as the optimizer, and has an initial learning rate of 0.0001, adopting a step learning rate adjustment strategy. We set the batch size to 8 and trained the model for a total of 50 epochs. To enable the model to fully extract the temporal correlations within the data, we extracted eight frames adjacent to the end-diastolic and end-systolic frames in each dataset (four frames before the key frame and three frames after the key frame) as the model’s input. To enhance the model’s robustness, we performed a series of data augmentations, including random horizontal flip, random rotation from −20° to 20°, random scaling from 0.9 to 1.1, random shifting from −0.0625 to 0.0625, and random adjustments to brightness and contrast from 0.8 to 1.2.

### 4.4. Experimental Results

#### 4.4.1. Comparison with Other Methods

**Figure 7:**
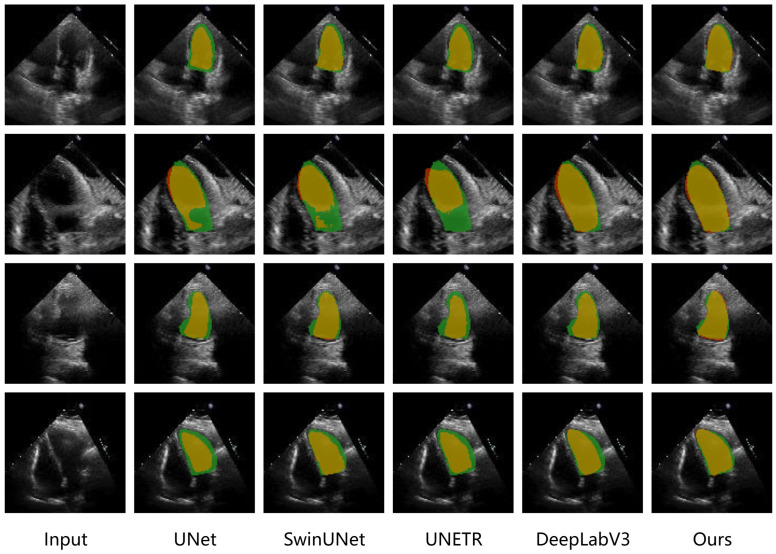
Visualization of the predictions from different methods on the EchoNet dataset. Green, red, and yellow regions represent ground truth, prediction, and overlapping regions, respectively.

We compared the performance of several typical models on the EchoNet dataset and the CAMUS dataset, including UNet [10], UNETR [16], SwinUNet [14], TransUNet [13], nnUNet [41], DeepLabV3 [28], Bi-DCNet [42], EchoGraphs [31] and MemSAM [43]. The results are shown in Table 1 and Table 2. The results on the two datasets are presented in Table 1 and Table 2, while Figure 7 illustrates the segmentation results of different models across multiple data samples. The experimental results demonstrate that, compared to the basic UNet architecture, incorporating Transformer self-attention into the network leads to varying degrees of performance improvement. Notably, the TransUNet model, which adds a Transformer after the encoder, yields the best results. This confirms that the global dependencies brought about by self-attention have a positive impact on echocardiographic segmentation. A comparison of the performance gains across the two datasets reveals that the effect of Transformer is more pronounced on the larger dataset. On the data-limited CAMUS dataset, nnUNet also exhibits excellent performance by adapting the network architecture and preprocessing strategies. On the other hand, DeepLabV3 uses dilated convolutions to expand the receptive field and designs a pyramid pooling module to integrate features. This demonstrates that expanding the receptive field to gather more contextual information effectively improves model performance in echocardiographic segmentation tasks. Furthermore, SwinUNet outperforms UNETR while maintaining a lower parameter count, which we attribute to the Swin Transformer’s shifted window approach that limits the receptive field of attention. This highlights the superiority of local attention over global attention in the semantic segmentation of echocardiography images. Our model leverages Vision RetNet to extract features and introduces spatial decay via the Manhattan distance, assigning greater weight to spatially closer features in self-attention calculations while preserving a global receptive field.

Additionally, the experimental outcomes of EchoGraphs and MemSAM suggest that emerging architectures such as graph neural networks and universal segmentation models hold promising potential in the field of echocardiographic segmentation.

#### 4.4.2. Ablation Study

To demonstrate the effectiveness of the Temporal Feature Fusion Module and the Vision Retentive Network encoder, we used UNETR as the baseline and replaced the decoder with the FPN. We made two modifications to the baseline architecture: adding the Temporal Feature Fusion Module before the encoder and replacing the Transformer encoder with the Vision RetNet encoder. The final results are shown in Table 3. It is observed that both the Temporal Feature Fusion Module and the Vision RetNet encoder improved the model’s segmentation performance, with the replacement of the Transformer encoder by the Vision RetNet encoder, yielding a more significant improvement of 0.52%. Notably, while the Temporal Feature Fusion Module had a limited impact on the baseline, it significantly enhanced the performance of the Vision RetNet encoder. This indicates that the introduction of explicit spatial priors by the Vision RetNet effectively improves the segmentation performance of echocardiography images, and the explicit spatial prior information also amplifies the impact of inter-frame correlations on segmentation performance.

**Figure 8:**
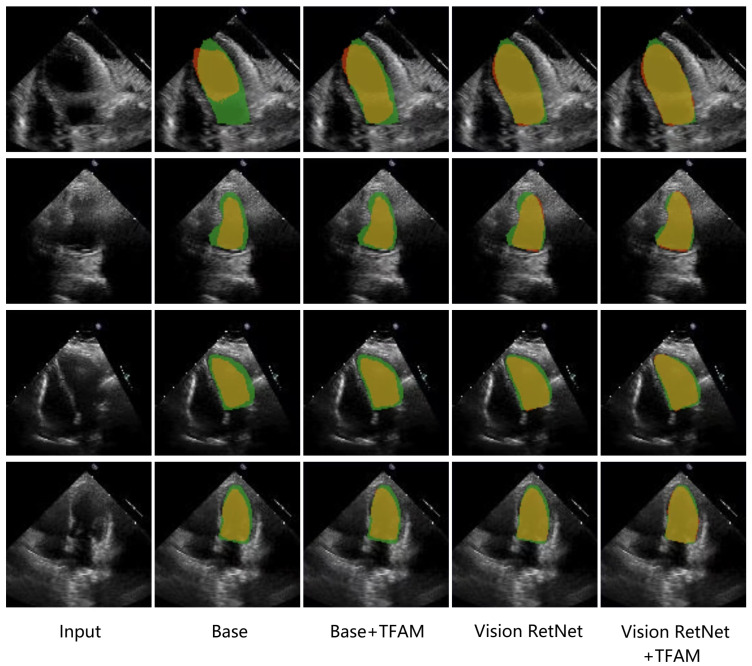
Visualization of the predictions from our model after modifying the configurations of TFFM and Vision RetNet on the EchoNet dataset. Green, red, and yellow regions represent ground truth, prediction, and overlapping regions, respectively.

Figure 8 presents the visual comparison results of the ablation experiments on the EchoNet dataset. In the second row, images are notably affected by speckle noise interference, while in the fourth row, the boundaries of the left ventricle are blurry. In the first and third rows, the shapes of the left ventricle are rotated due to the acquisition view. It can be observed that despite these interferences, our proposed model can still accurately identify the contours of the left ventricle.

Table 4 investigates the impact of different self-attention architectures and the two submodules (TCSA and CA) within the TFFM module on segmentation performance. A comparison of the segmentation performance between the three self-attention-augmented architectures—two Transformer-based models and Vision RetNet—and UNet reveals that global dependencies can enhance the model’s segmentation capabilities. Furthermore, although all three architectures show some degree of performance improvement compared to UNet, TransUNet demonstrates a more pronounced performance gain than UNETR. We attribute this difference to the architectural distinctions between TransUNet and UNETR. In the encoder stage, TransUNet first extracts features through a convolutional neural network and then applies Transformer layers to deeper features, learning global dependencies, while UNETR directly utilizes Transformer layers for feature extraction. This highlights the critical role of local features during the feature extraction phase. In this regard, Vision RetNet introduces explicit spatial priors, bestowing greater relevance to spatially proximate features, thereby enabling the self-attention mechanism to enhance its sensitivity to local features, resulting in superior performance.

In addition to exploring the effects of different self-attention architectures, we further investigate the individual contributions of the two submodules within the Temporal Feature Fusion Module (TFFM) to segmentation performance. The TFFM module consists of two parts: the first, temporal–channel self-attention (TCSA), primarily captures inter-frame correlations across multiple frames of images; the second, the channel feature aggregation module, focuses on extracting complementary interaction information along the channel dimension to aggregate features. We applied both submodules to the three self-attention architectures and observed that, regardless of the self-attention architecture used, the temporal–channel self-attention module significantly enhances segmentation performance, especially in the Vision RetNet architecture. This further corroborates the positive impact of inter-frame correlations in echocardiography on image segmentation. The role of the channel aggregation module is mainly to aggregate features along the time–channel dimension and extract complementary features. The extraction of complementary features is contingent upon the model’s ability to effectively learn the inter-frame correlations in echocardiography. Therefore, this submodule serves a primarily auxiliary role.

To validate the impact of decomposing MaSA into 1D self-attention, we replaced the decomposed MaSA in the third stage of the model with the original MaSA and compared the segmentation performance and computational cost for both settings. The results, as shown in the Table 5, indicate that while the segmentation performance remains virtually unchanged after decomposing MaSA, the computational cost is significantly reduced. This suggests that the MaSA decomposition operation can effectively reduce computational load with minimal impact on segmentation performance.

## 5. Conclusions

In this study, we propose a novel echocardiography segmentation model that integrates features from multiple echocardiography images to enhance the segmentation of ED and ES frames. The model employs Vision RetNet as the encoder, incorporating explicit spatial priors through the Manhattan distance. Our experimental results demonstrate that, benefiting from its perception of explicit spatial priors and inter-frame correlations, our model exhibits greater robustness to ambiguity in echocardiograms, delivering competitive performance. This marks the first application of Vision RetNet to echocardiography images, paving the way for future research exploring its use across various modalities of medical imaging data.

## Figures and Tables

**Table 1:** Comparison of different models on the EchoNet-Dynamic dataset.

Model	Architecture	DSC (%) ↑	HD95 (Pixels) ↓	Params (MB)
U-Net	CNN	91.36	4.98	9.5
UNETR	CNN+Transformer	92.43	3.06	87.5
SwinUNet	Transformer	92.67	2.92	27.1
TransUNet	CNN+Transformer	92.93	2.84	105.1
DeepLabV3	CNN	92.68	2.83	39.6
nnUNet	CNN	92.86	-	**7.37**
Bi-DCNet	CNN	92.28	-	-
EchoGraphs	CNN+GCN	92.10	-	27.1
MemSAM	Segment Anything	92.78	4.57	-
Ours	CNN+RetNet	**93.17**	**2.72**	35.2

The ↑ indicates that the higher this metric, the stronger the model’s segmentation performance, while the ↓ indicates that the lower this metric, the stronger the model’s segmentation performance. The same applies to the table below.

**Table 2:** Comparison of different models on the CAMUS dataset.

Model	Architecture	DSC (%) ↑	HD95 (Pixels) ↓
U-Net	CNN	92.17	7.12
UNETR	CNN+Transformer	92.45	7.37
SwinUNet	Transformer	92.56	6.82
TransUNet	CNN+Transformer	93.42	6.07
DeepLabV3	CNN	92.91	6.57
nnUNet	CNN	93.33	-
MemSAM	Segment Anything	93.31	**4.57**
Ours	CNN+RetNet	**93.70**	5.81

**Table 3:** Ablation of two components (TFFM and Vision RetNet) of the model we propose on the EchoNet dataset.

Setting	DSC (%) ↑	HD95 (Pixels) ↓
Baseline(UNETR)	92.43	3.06
Baseline+TFFM	92.49	3.04
Baseline+Vision RetNet	92.95	2.72
Vision RetNet+TFFM	93.17	2.72

**Table 4:** Ablation of different types of self-attention architecture and the submodules of TFFM on the CAMUS dataset.

Setting	DSC (%) ↑	HD95 (Pixels) ↓
UNet (no self-attention)	92.17	7.12
TransUNet	93.42	6.07
TransUNet+TCSA	93.50	6.07
TransUNet+CA	93.46	6.07
TransUNet+TFFM	93.53	6.06
UNETR	92.45	7.37
UNETR+TCSA	92.53	7.37
UNETR+CA	92.48	7.37
UNETR+TFFM	92.55	7.37
Vision RetNet	93.40	5.81
Vision RetNet+TCSA	93.61	5.80
Vision RetNet+CA	93.45	5.81
Vision RetNet+TFFM	93.70	5.81

**Table 5:** Comparison of decomposed MaSA and original MaSA on the CAMUS dataset.

3rd Stage	DSC (%) ↑	FLOPs (G)
Original MaSA	93.63	412.6
Decomposed MaSA	93.60	340.1

## Data Availability

The EchoNet-Dynamic dataset is publicly available at https://echonet.github.io/dynamic/ (accessed on 5 March 2023).

## Abbreviations

The following abbreviations are used in this manuscript:

CVD	Cardiovascular Disease
LV	Left Ventricle
ED	End-Diastole
ES	End-Systole
CNN	Convolutional Neural Network
CT	Computed Tomography
MRI	Magnetic Resonance Imaging
RetNet	Retentive Network
FPN	Feature Pyramid Network
LSTM	Long Short-Term-Memory
TFFM	Temporal Feature Fusion Module
Conv	Convolution
DWConv	Depth-Wise Convolution
TCSA	Temporal–Channel Self-Attention
CA	Channel Aggregation
MaSA	Manhattan Self-Attention
2D	2-Dimensional
1D	1-Dimensional
DSC	Dice Similarity Coefficient
HD95	The 95th percentile of the Hausdorff Distance
GCN	Graph Convolution Network

## Appendix A. Python Environment of Our Method

All the code was compiled under Python 3.8.19, using PyCharm as the IDE. All computations were executed on RTX 3090 GPU (NVIDIA, Santa Clara, CA, USA). More details about the versions of Python packages are listed below.

**Table A1:** Versions of Python packages.

Python Packages	Versions
Python	3.8.19
albumentations	1.3.0
eniops	0.7.0
Medpy	0.5.2
monai	1.4.0
mmcv	2.2.0
mmengine	0.10.2
mmsegmentation	1.2.2
numpy	1.24.4
pandas	2.0.3
pillow	10.4.0
scikit-image	0.23.2
scikit-learn	1.5.0
scipy	1.13.1
SimpleITK	2.3.0
torch	2.4.0
torchvision	0.19.0
